# Supplementary material for: A 13CO2 Enrichment Experiment to Study the Synthesis Pathways of Polyunsaturated Fatty Acids of the Haptophyte Tisochrysis lutea
Source: Mar Drugs. 2021 Dec 24;20(1):22. doi: 10.3390/md20010022 (PMC8779623; doi:10.3390/md20010022)
Supplement: Supplementary file 1 [file marinedrugs-20-00022-s001.zip › SUPPLEMENTARY FILES-REMIZE et al.pdf]

SUPPLEMENTARY FILES

Table S1: Cellular parameters of *Tisochrysis lutea* (morphology (FSC and SSC), viability (FL1 - SYTOX), and chlorophyll content (FL3) using flow cytometry analysis (Mean ± SD of the 3 balloons). Values for SYTOX are in %, values for FL3/SSC/FSC in arbitrary unit (a.u).

	**FL1 – SYTOX ALIVE**			**FL3**			**SSC**			**FSC**		
**0**	98	±	1	134	±	9	52	±	7	165	±	4
**0.5**	95	±	2	139	±	10	49	±	6	165	±	6
**1**	95	±	1	140	±	9	44	±	2	167	±	5
**2**	94	±	0	134	±	11	49	±	10	168	±	4
**3**	95	±	3	134	±	11	49	±	11	169	±	4
**4**	95	±	2	134	±	11	44	±	8	170	±	5
**6**	95	±	2	134	±	11	47	±	10	170	±	2
**8**	94	±	2	134	±	11	52	±	4	169	±	2
**10**	93	±	2	135	±	10	46	±	5	171	±	3
**12**	96	±	1	136	±	9	44	±	4	171	±	2
**14**	97	±	1	137	±	11	44	±	6	171	±	2
**16**	97	±	1	137	±	12	44	±	3	171	±	3
**18**	97	±	1	136	±	13	44	±	3	173	±	4
**20**	96	±	2	136	±	13	46	±	4	173	±	3
**22**	97	±	1	136	±	13	42	±	6	174	±	3
**24**	97	±	1	137	±	12	42	±	5	175	±	2

Table S5: Mean ratio of atomic enrichment (AE) for pairs of FA (FA_A vs FA_B) in the neutral lipids (NL) (mean ± SD, n=9 sampling dates t_8 to t_{24}) for the two enriched balloons (Tl1, Tl2, Tl = *Tisochrysis lutea*). If the ratio is equal to or close to 1, A and B are assumed at equilibrium, and B is synthesized quickly from A; if the ratio is below 1, the transformation of B from A is possible but slow. Finally, if the ratio is above 1, A is not a main precursor of B, which has to be synthesized by a different pathway.

	**Neutral lipids**			
	Tl1		Tl2	
Fatty acid B/Fatty acid A	Mean	SD	Mean	SD
18:5n-3/18:4n-3	-	-	-	-
20:5n-3/18:5n-3	3.58	1.78	3.33	1.40
22:5n-3/20:5n-3	0.92	0.14	0.64	0.12
22:6n-3/22:5n-3	0.55	0.17	0.79	0.14
22:6n-3/20:5n-3	0.49	0.11	0.50	0.11
22:6n-3/22:5n-6	0.49	0.15	0.65	0.15

Table S6: List of the potential candidate protein sequences involved in *Tisochrysis lutea* PKS synthesis pathway. The suspected function of each protein has been assumed using the NCBI Conserved Domain Database (CDD) (Marchler-Bauer et al., 2017) by identifying the role of each domain recognized in the sequence. In columns KS/KR/DH/ER are written the number of domain corresponding to these functions in the studied sequences. **ACS**: Acetyl-CoA synthetase, **A_NRPS**: Adenylation domain of the non-ribosomal peptide synthetase (**NRPS**), **Croto**: crotonase/enoyl-CoA hydratase, **EntF**: Enterobactin non-ribosomal peptide synthetase or thioesterase domain of Type I PKS, **FAAL**: Fatty Acyl-AMP ligase, **GrsT**: alpha/beta hydrolase, **HM**: hydroxymethylglutaryl-CoA synthase, **MT**: methyltransferase, **PP**: PhosphoPantetheine-binding (= “swinging arm”), **Sulf**: sulfotransferase, **Thio**: thioesterase

NAME	4 DOMAINS	Cluster	KS	KR	DH	ER	OTHER DOMAINS	SUSPECTED FUNCTION
**TISO_16054**	No		0	0	1	0	No	3-hydroxyacyl-ACP dehydratase
**TISO_09404**	No		1	0	0	0	No	β-ketoacyl-ACP synthase II
**TISO_19207**	No		1	0	0	0	No	3-ketoacyl-CoA synthase
**TISO_09037**	No		0	1	0	0	No	β-ketoacyl-ACP reductase II
**TISO_04539**	Yes		7	1	1	1	PP+FAAL	Supposed involved in complex lipid synthesis
**TISO_06404**	Yes		4	3	2	1	PP+Thio	Thioesterase
**TISO_06537**	Yes		4	5	4	3	PP+Sulf+EntF	Thioesterase / Sulfotransferase
**TISO_08010**	No		1	0	0	0	No	β-ketoacyl-ACP synthase II
**TISO_08047**	Yes		17	18	16	14	PP+MT+Thio	Thioesterase / Methyltransferase
**TISO_11097**	Yes		6	5	4	4	PP+EntF	Peptide synthesis
**TISO_14040**	No		1	0	0	0	No	β-ketoacyl-ACP synthase II
**TISO_14962**	Yes	Cluster 1	7	8	5	4	PP+MT+Thio	Methyltransferase / Thioesterase
**TISO_14968**	Yes	Cluster 1	2	3	2	3	PP	FA synthesis
**TISO_14973**	No	Cluster 1	1	0	1	0	PP	Ketoacyl-synthase for n-3 PUFA
**TISO_14975**	Yes	Cluster 1	1	2	1	2	PP	FA synthesis
**TISO_14977**	Yes	Cluster 1	4	2	2	3	PP + ACS	Acetyl-CoA synthetase / FA synthesis
**TISO_15188**	No		1	1	0	0	PP+GrsT+A_NRPS	Peptide synthesis
**TISO_16495**	Yes		6	9	5	4	PP+FAAL+Sulf+EntF+Thio	Thioesterase / Sulfotransferase
**TISO_27353**	Yes	Cluster 2	2	1	1	1	PP+EntF	Thioesterase / Peptide synthesis
**TISO_27354**	No	Cluster 2	2	0	0	0	PP+FAAL	Supposed involved in complex lipid synthesis
**TISO_30977**	No		5	2	2	0	PP+MT+Croto+HM	Complex lipid synthesis
**TISO_37254**	No	Cluster 3	1	2	0	1	PP+Sulf+EntF	Thioesterase / Sulfotransferase
**TISO_37256**	No	Cluster 3	1	0	0	0	No	Claisen condensation
**TISO_37258**	No	Cluster 3	1	0	1	0	No	Claisen condensation / Dehydratase
**TISO_37259**	No	Cluster 3	0	0	0	0	PP	Phosphopantetheine binding
**TISO_37260**	Yes	Cluster 3	7	6	5	3	PP+FAAL	Supposed involved in complex lipid synthesis
**TISO_37578**	No	Cluster 4	1	0	0	0	No	Claisen condensation
**TISO_37579**	Yes	Cluster 4	3	2	2	1	PP	FA synthesis
**TISO_37581**	No	Cluster 4	1	0	0	0	PP	Claisen condensation / PP-binding
**TISO_37631**	Yes		4	3	4	3	PP	FA synthesis

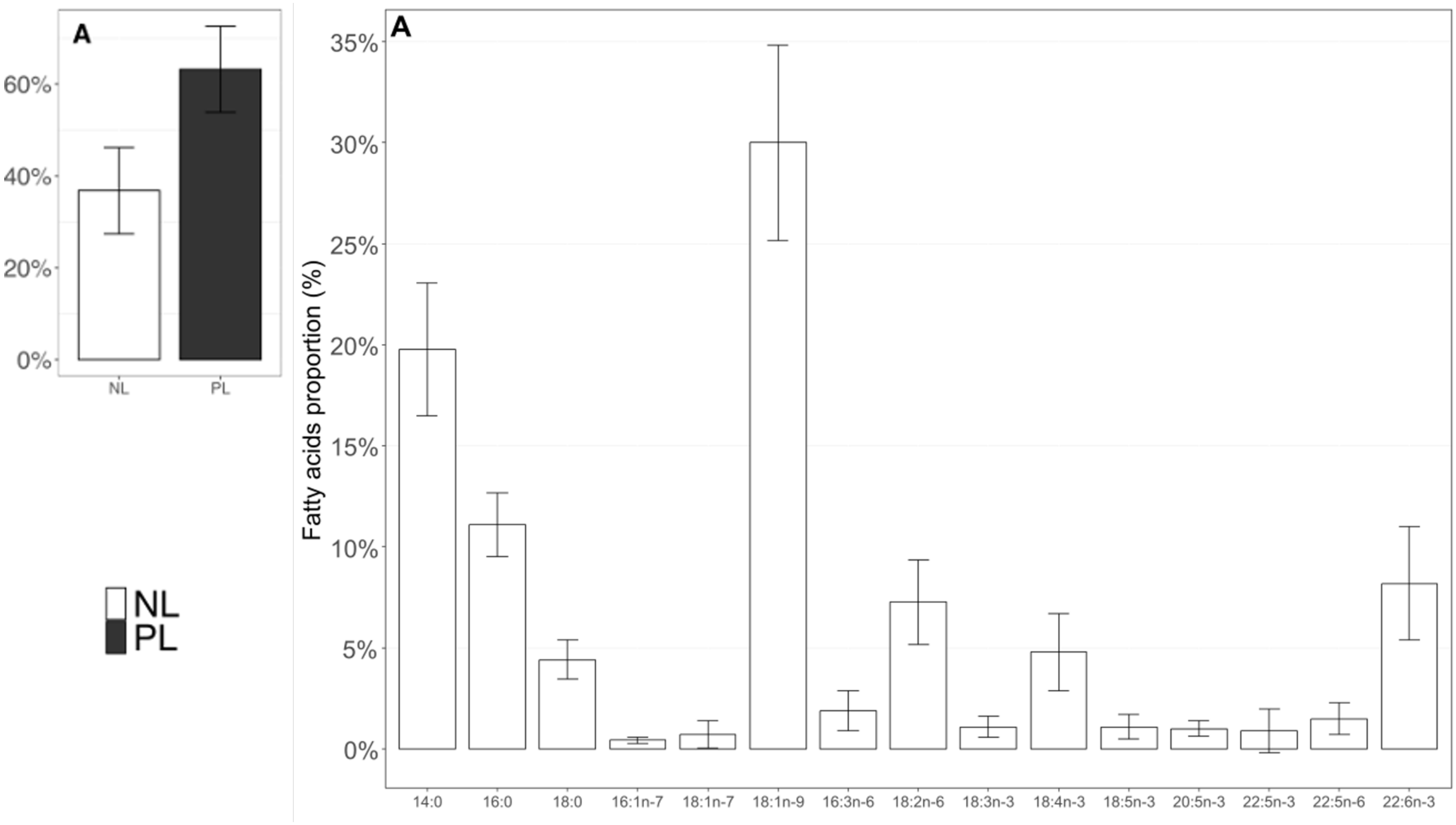

Figure S1: Proportions (%) of NL vs PL [A] and proportions (%) in average over the 24 hours of fifteen fatty acids in the NL fraction [B]

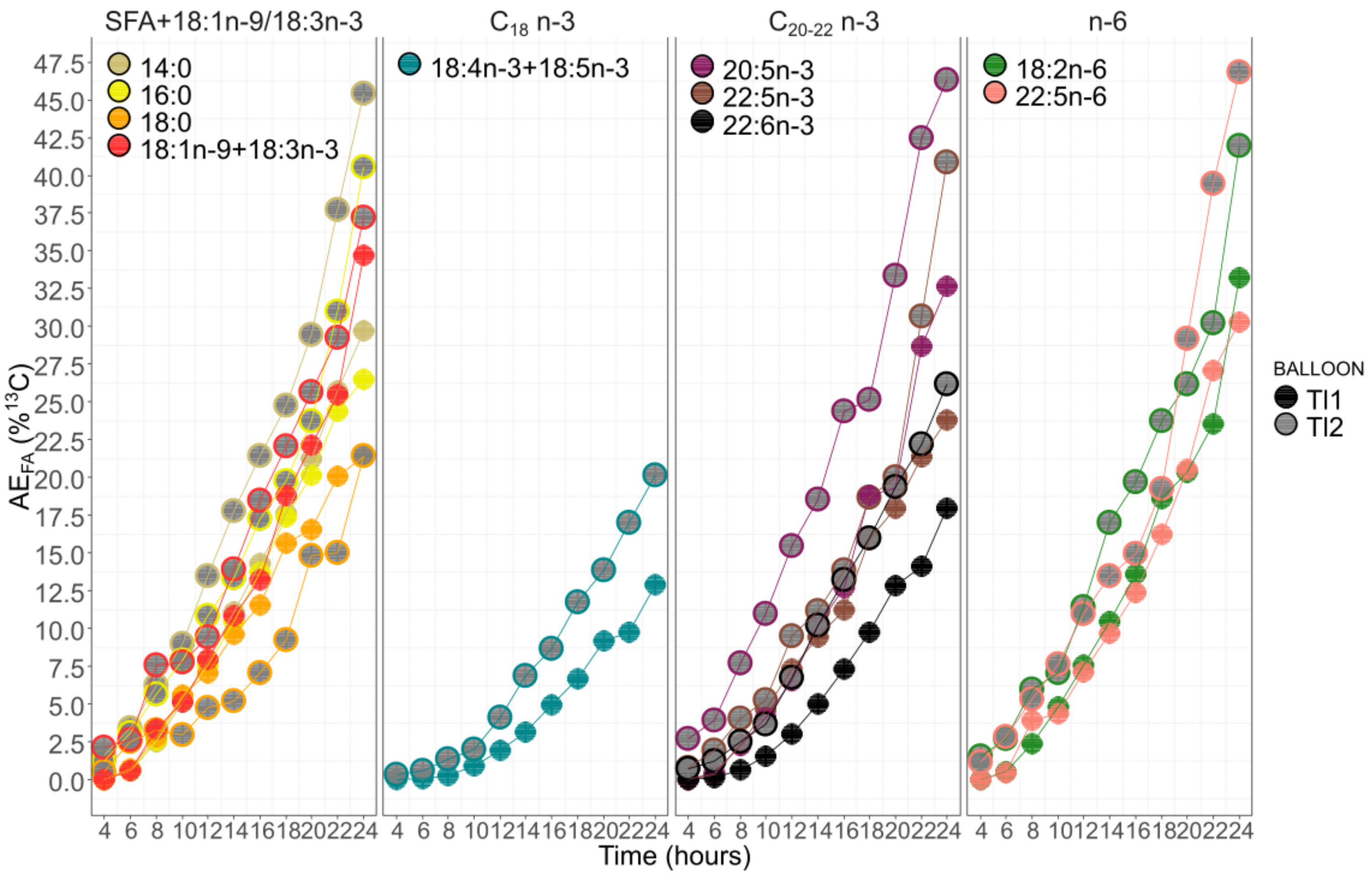

Figure S2: Atomic enrichment of 11 main fatty acids in the polar lipid (NL) fraction during a 24h ^{13}C labelling experiment. Tl: *Tisochrysis lutea*.
